# On-the-road driving performance and driving-related skills in older untreated insomnia patients and chronic users of hypnotics

**DOI:** 10.1007/s00213-014-3455-z

**Published:** 2014-02-02

**Authors:** T. R. M. Leufkens, J. G. Ramaekers, A. W. de Weerd, W. J. Riedel, A. Vermeeren

**Affiliations:** 1 Philips Group Innovation–Research, Division Information and Cognition, Department of Brain, Body and Behavior, High Tech Campus 34, 5656 AE Eindhoven, The Netherlands; 2 Experimental Psychopharmacology Unit, Department of Neuropsychology and Psychopharmacology, Faculty of Psychology and Neuroscience, Maastricht University, Maastricht, The Netherlands; 3 Department of Clinical Neurophysiology and Sleep Centre SEIN, Zwolle, The Netherlands

**Keywords:** On-the-road driving, Insomnia, Hypnotics, Cognition, Elderly

## Abstract

**Rationale:**

Many older adults report sleep problems and use of hypnotics. Several studies have shown that hypnotics can have acute adverse effects on driving the next morning. It is unclear however whether driving of chronic hypnotic users is impaired. Therapeutic effects on insomnia and development of tolerance may reduce the residual effects on driving.

**Objectives:**

The present study aimed to compare actual driving performance and driving-related skills of chronic hypnotic users to good sleepers. To determine whether insomnia itself affects driving performance, driving and driving-related skills were compared between insomnia patients who do not or infrequently use hypnotics and good sleepers.

**Methods:**

Twenty-two frequent users of hypnotics (using hypnotics ≥4 nights per week for more than 3 months), 20 infrequent users (using hypnotics ≤3 nights per week), and 21 healthy, age-matched controls participated in this study. On the night before testing, all subjects were hospitalized for an 8-h sleep recorded by polysomnography. Frequent hypnotic users used their regular medication at bedtime (2330 hours), while infrequent users and controls received no medication. Cognitive performance (word learning, digit span, tracking, divided attention, vigilance, and inhibitory control) was assessed 8.5 h and driving performance between 10 and 11 h after bedtime and dosing.

**Results:**

Polysomnographic recordings did not significantly differ between the groups, but the insomnia patients, treated or untreated, still reported subjective sleep complaints. Results show no differences in driving performance and driving-related skills between both groups of insomnia patients and controls.

**Conclusions:**

Driving performance in chronic users of hypnotics and untreated insomnia patients is not impaired. For chronic users, this may be due to prescription of relatively safe drugs and low doses. For untreated insomniacs, this corroborates previous findings showing an absence of neuropsychological deficits in this group of patients.

## Introduction

Approximately one third of the general adult population suffers from insomnia, reporting difficulties in initiating sleep or in maintaining sleep, and feelings of nonrestorative sleep (Ohayon 2002; Morin et al. 2006). Although non-pharmacological strategies, such as cognitive behavioral therapy, are increasingly being implemented in the treatment of insomnia, pharmacotherapy is still the most frequently used treatment (Morin et al. 2007). The primary choice of sleep-enhancing medication is sedative hypnotics, such as benzodiazepines and the newer benzodiazepine receptor agonists zopiclone, zolpidem, and zaleplon (Verster et al. 2007; Lader 2011).

Ideally, a hypnotic should improve sleep, but be free from residual sedative effects after arising. It is known, however, that a number of hypnotics that are currently prescribed can produce next-day residual sedation, depending on type of hypnotic, dose, time after administration, and frequency of dosing (Vermeeren 2004). This may lead to impairment of a wide range of cognitive abilities and can have serious consequences for daily activities, such as driving a car. In epidemiological studies, for example, it has been shown that use of hypnotics is related to an increased risk of becoming involved in traffic and occupational accidents (Ray et al. 1992; Hemmelgarn et al. 1997; Barbone et al. 1998; Neutel 1998; Dubois et al. 2008; Dassanayake et al. 2011). Experimental studies assessing actual driving performance after administration of hypnotics confirm these data by showing residual driving impairment in the morning after dosing (Volkerts and O’Hanlon 1988; Vermeeren 1995; Vermeeren et al. 1998, 2002; Verster et al. 2002; Leufkens et al. 2009).

In order to select the safest alternative among the available hypnotics, patients and prescribing physicians should be informed about the possible impairing effects of hypnotics. To date, information of the residual effects on driving performance is mainly derived from experimental studies conducted with young, healthy, hypnotic-naïve volunteers after a single night of treatment. Investigating the residual effects in this population leaves two important issues unanswered. First, it may be possible that the effects of hypnotics interact with insomnia in such a way that they are experienced differently between insomniacs and healthy, young volunteers. Secondly, the majority of insomniacs use hypnotics chronically, which may induce tolerance to their residual effects. As a consequence, the impairing effects may be less pronounced in insomniacs than in healthy volunteers.

Untreated insomniacs report reduced performance in daily life routines, which may have serious detrimental consequences (Chambers and Keller 1993; Varkevisser and Kerkhof 2005; Sarsours et al. 2011; Roth et al. 2011). It can, therefore, be expected that daytime performance after hypnotic-induced sleep is improved in insomniacs. Healthy volunteers, on the other hand, cannot benefit from hypnotic treatment as their performance is already optimal. Impairment observed due to hypnotic sedation in healthy volunteers may therefore be an overestimation of the net effects of hypnotics in patients. However, most experimental studies examining cognitive and psychomotor performance showed that daytime functioning in untreated insomnia patients was not (Riedel and Lichstein 2000; Fulda and Schulz 2001) or only minimally impaired (Shekleton et al. 2010; Bastien 2011; Fortier-Brochu et al. 2012).

Whereas experimental studies have failed to demonstrate impaired daytime functioning, it has been shown in a cross-sectional epidemiological study that difficulties in sleeping are associated with an increased risk in occupational fatal accidents (Akerstedt et al. 2002). More recently, in a study investigating the relationship between health-related complaints and crash involvement risk, it was found that sleep disturbances were associated with an elevated risk of becoming involved in car accidents (Sagberg 2006). The differences in findings of experimental and epidemiological research may be explained by possible limitations related to the type of research. Epidemiological studies may not have been able to control for other factors contributing to impaired daytime functioning. For example, insomnia is strongly associated with disorders such as depression and anxiety (Stewart et al. 2006). These disorders have been shown to affect daily functions as well (Kindermann and Brown 1997; Kizilbash et al. 2002; Wingen et al. 2006) and may have influenced the results. The absence of objective impairment in experimental studies is mainly explained by a lack of laboratory tests demanding high effort (Vignola et al. 2000; Altena et al. 2008; Edinger et al. 2008). Most performance tasks are of short duration and insomniacs may relatively easy be able to maintain high-level performance during testing (Varkevisser and Kerkhof 2005).

The second issue that has not yet been clarified in experimental designs using healthy young volunteers is whether residual effects of hypnotics are still present in insomniacs who chronically use hypnotics. Although it is not recommended to use hypnotics for periods longer than 4 weeks, a majority of insomnia patients are treated for prolonged periods (Ashton 2005; Paterniti et al. 2002). This may induce the development of tolerance to the residual effects of hypnotics (Bateson 2002). The impairing effects in hypnotic-naïve volunteers might therefore be larger than the actual effects on performance of insomniacs chronically using hypnotics. For example, a recent experimental study demonstrated that performance of chronic users of hypnotics was comparable to that of untreated insomnia patients and self-defined good sleepers (Vignola et al. 2000).

In summary, it is unclear whether driving performance of chronic users of hypnotics is impaired. The primary objective of the present study was therefore to compare actual driving performance and driving-related skills of chronic users of hypnotics to good sleepers. In case no difference in performance is found between these groups, this might be due to tolerance to the adverse effects of hypnotics, or the therapeutic effects of hypnotics on insomnia and performance, or both. To determine whether insomnia itself has adverse effects on driving performance, the secondary objective was to compare driving and driving-related skills between insomnia patients who do not or infrequently use hypnotics and good sleepers.

## Methods

### Subjects

A total of 42 insomnia patients and 21 healthy controls were recruited through a network of local general practitioners in the region of Maastricht, The Netherlands (Regionaal Netwerk Huisartsen), and by advertisement in local newspapers. All participants had to meet the following inclusion criteria: aged between 50 and 75 years; possession of a valid driving license for at least 3 years; average driving experience of at least 3,000 km per year over the last 3 years; good health based on a pre-study physical examination, medical history, vital signs, electrocardiogram, blood biochemistry, hematology, serology, and urinalysis. Exclusion criteria were history of drug or alcohol abuse; presence of a significant medical, neurological, psychiatric disorder, or sleep disorder other than insomnia; chronic use of medication that affects driving performance, except hypnotics; drinking more than 6 cups of coffee per day; drinking more than 21 units of alcohol per week; smoking more than 10 cigarettes per day; and body mass index outside the range of 19 to 30 kg/m^2^.

Insomnia patients had to meet the inclusion criteria for primary insomnia according to DSM-IV (American Psychiatric Association 1994): (1) subjective complaints of insomnia, defined as difficulties initiating sleep (sleep latency >30 min) and/or maintaining sleep (awakenings >30 min); (2) duration of more than 1 month; (3) the sleep disturbance causes clinically significant distress or impairment; (4) insomnia does not occur exclusively during the course of a mental disorder; and (5) insomnia is not due to another medical or sleep disorder or effects of medication or drug abuse.

Volunteers were screened by a telephone interview, questionnaires, and a physical examination. Insomnia patients’ sleep complaints were evaluated by a trained psychologist using Dutch versions of the Pittsburgh Sleep Quality Index (PSQI) (Buysse et al. 1989), the Sleep Wake Experience List (SWEL) (van Diest et al. 1989), and the Groningen Sleep Quality Scale (GSQS) (Mulder-Hajonides van der Meulen 1981). The GSQS provides a score between 0 and 14 representing a number of sleep complaints. In addition, subjects completed a sleep log for 14 days, providing daily information about estimated sleep and wake times, and sleep quality using the GSQS (Mulder-Hajonides van der Meulen 1981). Major psychopathology was screened using the Symptom Checklist 90 Revised (SCL90-R) (Derogatis 1983), the Beck Depression Inventory (BDI) (Beck et al. 1961), the State-Trait Anxiety Inventory (STAI) (Spielberger et al. 1983), and the Multidimensional Fatigue Inventory (Smets et al. 1995).

Insomnia patients were assigned to one of two groups depending on the frequency and duration of their use of hypnotic drugs (benzodiazepine, zopiclone, or zolpidem). Patients were assigned to a “frequent users” group when they used a hypnotic for at least four nights per week and longer than 3 months (*n* = 22). Patients not using hypnotics or using hypnotics less than or equal to 3 days per week were assigned to the “infrequent users” group (*n* = 20).

The study was conducted in accordance with the code of ethics on human experimentation established by the World Medical Association’s Declaration of Helsinki (1964) and amended in Edinburgh (2000). The protocol was approved by the medical ethics committee of Maastricht University and University Hospital of Maastricht. Subjects were explained the aims, methods, and potential hazards of the study and they signed a written informed consent prior to any study-related assessments.

### Assessments

#### Driving performance

Driving performance was assessed using two standardized driving tests developed to measure different aspects of driving performance. The primary test is the Highway Driving Test (O’Hanlon 1984) which measures road tracking performance. Performance in this test is mainly determined by the delay lag between sensory information, execution of motor reaction, and the vehicle’s dynamic response. In this test, subjects operate a specially instrumented vehicle over a 100-km (61-mi) primary highway circuit, accompanied by a licensed driving instructor having access to dual controls. The subjects’ task is to maintain a constant speed of 95 km/h (58 mi/h) and a steady lateral position between the delineated boundaries of the slower traffic lane. The vehicle speed and lateral position are continuously recorded. These signals are edited offline to remove data recorded during overtaking maneuvers or disturbances caused by roadway or traffic situations. The remaining data are then used to calculate means and standard deviations of lateral position and speed. Standard deviation of lateral position (SDLP in centimeters) is the primary outcome variable. SDLP is a measure of road tracking error or “weaving.” The test duration is approximately 1 h.

SDLP is an extremely reliable index of individual driving performance and has proven sensitive to many sedating drugs (O’Hanlon and Ramaekers 1995; O’Hanlon et al. 1995; Ramaekers 2003; Vermeeren 2004; Verster 2004). Test–retest reliability ranges between 0.7 and 0.9 in individual studies and is on average 0.85. The test was calibrated for the effects of alcohol in a closed circuit study wherein 24 social drinkers were tested sober and after controlled drinking to raise blood alcohol concentrations in steps of 0.3 g/L to a maximum of 1.2 g/L (Louwerens et al. 1987). In line with the relationship between blood alcohol concentration (BAC) and accident risk as estimated in a large epidemiological study by Borkenstein (1974), the relationship between BAC and SDLP was shown to be an exponential function. Based on this relationship, BACs of 0.5, 0.8, and 1.0 g/L were associated with mean changes in SDLP of 2.4, 4.2, and 5.1 cm, respectively. The increase in SDLP caused by 0.5 g/L alcohol is considered clinically meaningful since accident risk has been demonstrated to increase significantly above this BAC in large epidemiological studies (Borkenstein 1974; Krüger et al. 1994). The average SDLP score of 219 healthy volunteers was 18.2 cm (SD = 3.1 cm) after placebo treatments in various recent studies (data on file). Based on these data, sample sizes of 20 subjects per group ensure 80 % power to detect a clinically meaningful difference between groups (with an alpha level of 0.05) in the primary variable of this study, SDLP.

The Car-Following Test (Brookhuis et al. 1994; Ramaekers and O’Hanlon 1994) measures changes in controlled information processing such as selective attention, stimulus interpretation and decision making, and speed of an adaptive motor response to events which are common in driving. In the test, two vehicles travel in tandem over a two-lane, undivided, secondary highway at 70 km/h (44 mi/h). An investigator drives the leading car and the subject, in the second car, is instructed to follow at a distance between 25 and 35 m. Subjects are further instructed to constantly attend the leading car since it may slow down or speed up at unpredictable times. They are required to follow the leading car’s speed movements, i.e., maintain the initial headway by matching the velocity of the car to the other’s. During the test, the speed of the leading car is automatically controlled by a modified “cruise control” system. At the beginning, it is set to maintain a constant speed of 70 km/h and, by activating a microprocessor, the investigator can start sinusoidal speed changes reaching amplitude of −10 km/h and returning to the starting level within 50 s. The maneuver is repeated six times. The leading car’s speed and signals indicating the beginning of the maneuver are transmitted via telemetry to be recorded in the following vehicle together with the following vehicle’s speed. Phase-delay converted to a measure of the subject’s average reaction time to the movement of the leading vehicle (RT, in second) is taken as the primary dependent variable in this test. A secondary measure in the Car-Following Test is Gain. It represents an amplification factor between the signals of the two cars. This will be larger than 1 when the subject overreacts to speed adaptations of the leading car. Test duration is 25 min.

#### Cognitive and psychomotor performance

Cognitive and psychomotor performance was assessed by tests for word learning, digit span, tracking, divided attention, sustained attention, and inhibitory control. Tests were selected based on their sensitivity to residual sedating effects of hypnotics or sleep disturbances, and their relationship to driving performance (Vermeeren 1995; Vermeeren et al. 1998, 2002; Fulda and Schulz 2001; Verster et al. 2002; Vermeeren 2004; Leufkens et al. 2009; Leufkens and Vermeeren 2009).

The Word Learning Test (Rey 1964) is a verbal memory test for the assessment of immediate recall, delayed recall, and recognition performance. Fifteen monosyllabic nouns are presented, and at the end of the sequence, the subject is asked to recall as many words as possible. This procedure is repeated five times, and after a delay of at least 30 min, the subject is again required to recall as many words as possible. At this trial, the nouns are not presented. Finally, a sequence of 30 monosyllabic nouns is presented, containing 15 nouns from the original set and 15 new nouns in random order. The subject has to indicate whether a noun originates from the old set or it is from a new set of nouns. The main performance parameters are Immediate Total Recall Score (number of immediately correctly recalled words), Delayed Recall Score (number of correctly recalled words after a 30-min delay), Recognition Score (number of correctly recognized words), and Recognition Response Time (in millisecond).

The Digit Span Forward and Backward is a subtest of the Wechsler Adult Intelligence Scale-Revised and measures the storage capacity of an individual’s working memory (Wechsler 1981). In this test, subjects are asked to repeat orally presented digits with increasing sequence length, either in forward or reverse order. There are two trials at each series length, and the test continues until both trials of a series length are failed. One point is awarded for each correct trial resulting in a Digit Span Forward Score and a Digit Span Backward Score.

The Critical Tracking Test (Jex et al. 1966) measures the ability to control an unstable signal in a tracking task. The signal deviates horizontally from a midpoint with increasing velocity and the subject has to compensate this signal deviation by moving a joystick in opposite direction. The velocity (lambda in radian/second) at which the subject loses control is measured. The test includes five trials of which the lowest and the highest score are discarded. The performance parameter is the average lambda (radian/second) of the remaining three scores, reflecting the critical tracking velocity.

The Divided Attention Task (Moskowitz 1973) measures the ability to divide attention between two simultaneously performed tasks. The first task is to perform the tracking test at a fixed level of difficulty, with velocity set at 50 % of the maximum score obtained after extensive training of the Critical Tracking Test. In the other task, the subject has to monitor 24 single digits that are presented in the four corners of the screen. The digits change asynchronously at 5-s intervals. The subjects are instructed to remove their foot from a pedal as rapidly as possible whenever the digit “2” appears. This signal occurs twice at every location, in random order, at intervals of 5 to 25 s.

In the Stop Signal Task (Logan et al. 1984), the concept of inhibitory control is defined as the ability to stop a pending thought or action and to begin another. The paradigm consists of two concurrent tasks, i.e., a go task (primary task) and a stop task (secondary task). The go signals (primary task stimuli) are two letters (“X” or “O”) presented one at a time in the center of a computer screen. Subjects are required to respond to each letter as quickly as possible by pressing on of two response buttons. Occasionally, a stop signal (secondary task stimulus) occurs during the test. Subjects are required to withhold any response in case a stop signal is presented. The stop signal consists of an auditory cue, i.e., a 1,000-Hz tone, that is presented for 100 ms. The interval at which the stop signal is presented is dependent from the subject’s own successful and unsuccessful inhibitions. By continuously monitoring the subject’s response, the Stop Signal Delay is adjusted producing the probability of responding [*p*(respond|signal)] equal to 0.50. Consequently, the stop signal reaction time is estimated by determining the mean of the inhibition function, which is then subtracted from the mean go RT. Task duration is 14 min.

The Psychomotor Vigilance Task (Dinges and Powell 1985) is based on a simple visual RT test. Subjects are required to respond to a visual stimulus presented at variable interval (2,000 to 10,000 ms) by pressing a button with the dominant hand. The visual stimulus is a counter turning on and incrementing from 0 to 60 s at 1-ms intervals. In response to the subject’s button press, the counter display stops incrementing, allowing the subject 1 s to read the RT before the counter restarts. If a response has not been made in 60 s, the clock resets and the counter restarts. The median reaction time and the number of lapses (i.e., response times >500 ms) were used as the main performance parameters. The test duration is 10 min.

#### Polysomnography

On nights before testing, sleep quality and duration were measured by polysomnography using montage including electroencephalogram, electrooculogram, and electromyogram. Sleep stages were visually assessed by experienced technicians according to standardized criteria (Iber et al. 2007). Technicians were only informed about the age and sex of the subjects, but were blinded to the group affiliation of the subjects. Parameters derived after analysis are sleep onset latency (in minute), wake after sleep onset (in minute), total sleep time (in minute), sleep efficiency (in percent), and number of awakenings.

#### Subjective evaluations

Upon arising, subjects completed the specific version of the Groningen Subjective Quality of Sleep questionnaire (GSQS) (Mulder-Hajonides van der Meulen 1981), providing a score between 0 and 14 representing a number of sleep complaints. In addition, subjects estimated sleep onset latency (in minute), number of awakenings, time awake before rising (in minute), and total sleep time (in minute).

Subjective evaluations of mood, sedation, and driving quality were assessed using a series of visual analogue scales (100 mm). The subjects were instructed to rate their subjective feelings using a 16-item mood scale which provides three-factor analytically defined summary scores for “alertness,” “contentedness,” and “calmness” (Bond 1974).

Subjective feelings of sleepiness were rated with the Karolinska Sleepiness Scale (Akerstedt and Gillberg 1990), ranging from 1 (extremely alert) to 9 (very sleepy, fighting sleep).

Subjects rated the degree of mental effort they had to put in driving performance with the Rating Scale Mental Effort (Zijlstra 1993). The scale is a visual analogue scale (150 mm) with additional verbal labels.

The driving instructors rated each subject’s driving quality and apparent sedation at the conclusion of the Highway Driving Test, using two 100 mm visual analogue scales ranging from “very bad” to “very good,” and from “not at all” to “completely,” respectively.

### Procedure

All subjects completed two nights of sleep evaluation and testing. The first night was a habituation and practice condition to familiarize the subjects with the sleeping facilities and polysomnographic and test procedures. The second night was for actual sleep and performance assessments. Within 10 days before their habituation night, the subjects were individually trained to perform the cognitive and psychomotor tests during two sessions of approximately 1.5 h.

A test condition started in the evening of day 1, when the subjects arrived at the site at approximately 1900 hours, and lasted until day 2, when they were discharged at approximately 114 hours. On arrival at the sleeping facility, the subjects rated their subjective feelings and subjective sleepiness. From 1930 until 2030 hours, they performed the first session of laboratory tests, comprising the Word Learning Test immediate and first delayed recall, the Critical Tracking Task, the Divided Attention Task, the Psychomotor Vigilance Task, the Stop Signal Task, and the Digit Span forward and backward. Hereafter, electrodes for polysomnographic recording were attached and subjects retired to bed at 2330 hours. Immediately preceding retiring, the subjects in the “frequent users” group ingested their own prescribed hypnotic, whereas the subjects in the “infrequent users” group and controls did not ingest medication.

The subjects were awakened at 0730 hours, and after arising, a light standardized breakfast was served. At 0800 hours, the subjects evaluated sleep quality and duration, and feelings of daytime sleepiness and alertness. Subsequently, they started the second session of laboratory tests, comprising the second delayed recall and word recognition parts of the Word Learning Test, the Critical Tracking Task, the Divided Attention Task, the Psychomotor Vigilance Task, and the Digit Span test. At 0900 hours, the subjects were transported to the Highway Driving Test which they performed between 0930 and 1030 hours. Upon completion, the subjects rated the mental effort it took to perform this driving test, and continued to perform a Car-Following Test. After this, the subjects returned to the testing facilities for removal of the electrodes and were discharged.

During participation, use of caffeine was prohibited from 8 h prior to arrival on test days, until discharge the next morning. Alcohol intake was not allowed from 24 h prior to each dosing until discharge. Smoking was prohibited from 1 h prior to bedtime until discharge.

### Statistical analysis

The primary parameter of the study was the SDLP (in centimeter). All performance-related parameters were analyzed for group differences at separate time points (evening, morning) using analysis of covariance (ANCOVA) with participant’s sex, age, and years of education as covariates. Significant (*p* < 0.05) main effects of group were further analyzed using three pairwise comparisons with LSD adjustment for multiple comparisons. Sleep parameters were analyzed for group differences using analysis of variance (ANOVA) with Welch test for unequal variances. Significant (*p* < 0.05) main effects of group were further analyzed using three pairwise comparisons with LSD adjustment for multiple comparisons. A natural log transformation was used before analysis of highly skewed variables. Tables show means and standard deviations of variances untransformed scores.

All statistical analyses were done by using the Statistical Package for the Social Sciences (SPSS) statistical program (version 21.0.0.1 for Windows; SPSS, Chicago, IL).

## Results

### Group differences for the screening variables

A total of 63 subjects (34 men, 29 women) completed the study. They had a mean (±SD) age of 61.6 (±5.1) years and an average education of 12 (±3) years. They had their driving license on average for 39 (±8) years and drove on average 9,965 (±7,848) km per year over the last 3 years. There were no significant differences between the three groups (Table 1).

**Table 1:** Means (±SD) of pre-study group characteristics

Variable	Frequent users (*n* = 22)	Infrequent users (*n* = 20)	Controls (*n* = 21)	Main effect of Group *F*
Sociodemographics				
Sex (M:F)	11:11	10:10	13:8	
Age (years)	62.1 (4.4)	60.8 (5.9)	61.7 (5.0)	0.40
Education (years)	12.3 (3.3)	10.9 (2.7)	13.2 (3.3)	2.67
Average annual mileage (km)	8,525 (6,269)	11,875 (11,534)	9,655 (4,157)	0.98
Driving license (years)	38.0 (8.3)	39.0 (8.4)	40.6 (5.9)	0.64
Sleep				
Pittsburgh Sleep Quality Index	12.6 (3.5)^a^	12.6 (2.6)^a^	2.4 (1.6)	100.2^***^
Groningen Subjective Quality of Sleep-general	9.4 (3.0)^a^	11.1 (1.7)^a,b^	1.0 (1.5)	126.1^***^
Sleep Wake Experience List^c^				
Sleep initiation problems	12^a^	10^a^	0	17.0^***^
Sleep maintenance problems	14^a^	13^a^	0	23.6^***^
Early morning awakenings	7^a^	5^a^	0	7.73^*^
Difficulty waking up	2	2	0	2.15
Tiredness upon waking up	3	2	0	2.91
Daytime sleepiness	5	3	1	2.84
Psychological				
Symptom Checklist 90-R				
Sleeping problems	9.6 (3.1)^a^	10.0 (2.5)^a^	3.3 (0.7)	52.6^***^
Depression	26.7 (9.7)^a^	27.1 (10.7)^a^	18.0 (3.0)	7.62^***^
Anxiety	15.8 (7.3)^a^	13.9 (4.8)	10.8 (1.3)	5.28^**^
Phobic anxiety	8.9 (4.5)	7.4 (1.0)	7.3 (0.8)	2.42
Psychoneuroticism	141.9 (37.1)^a^	137.9 (39.6)^a^	103.0 (13.1)	9.37^***^
Somatization	20.5 (7.9)^a^	19.1 (5.9)^a^	14.2 (1.8)	6.80^**^
Cognitive insufficiency	16.1 (6.3)^a^	15.8 (8.1)^a^	11.0 (1.8)	4.78^*^
Interpersonal sensitivity	25.8 (6.5)	24.8 (8.4)	21.7 (5.6)	2.11
Hostility	7.4 (1.6)	7.9 (2.8)	6.8 (1.3)	1.41
Beck Depression Inventory	8.6 (5.0)^a^	8.6 (6.6)^a^	2.8 (3.0)	9.38^***^
State-Trait Anxiety Inventory				
State anxiety	35.3 (10.1)^a^	41.9 (10.8)^a^	27.1 (6.2)	10.03^***^
Trait anxiety	38.4 (10.9)^a^	41.4 (12.8)^a^	27.2 (5.7)	9.30^***^
Multidimensional Fatigue Inventory				
General fatigue	11.8 (3.7)^a^	11.9 (3.7)^a^	6.8 (2.5)	16.4^***^
Physical fatigue	10.9 (3.5)^a^	10.6 (3.5)^a^	6.6 (2.3)	12.2^***^
Mental fatigue	11.5 (3.9)^a^	10.8 (3.5)^a^	7.0 (2.4)	11.2^***^
Reduced motivation	9.8 (3.6)^a^	9.6 (4.2)^a^	6.7 (2.5)	5.29^**^
Reduced activity	10.1 (4.2)^a^	10.0 (3.2)	7.4 (2.6)	4.03^*^

^*^ *p* < 0.05; ^**^ *p* < 0.01; ^***^ *p* < 0.001

^a^Significant difference with control group (*p* < 0.05; post hoc analysis with Bonferroni correction)

^b^Significant difference between frequent and in frequent users (*p* < 0.05; post hoc analysis with Bonferroni correction)

^c^Values for each variable are the frequencies in each group, and statistics represents results of Chi-square test

Evaluation of sleep at home differed significantly between groups. As shown in Table 1, sleep quality was poorer in both insomnia groups as compared to controls, as indicated by significantly higher scores on the PSQI, GSQS-general, and the sleep subscale of the SCL90-R (*p* < 0.001). There were no differences in mean PSQI, GSQS, and SCL90-R sleep scores between the insomnia groups. Scores on the SWEL showed that sleep complaints were most frequently classified by patients as sleep initiation and sleep maintenance problems, and occasionally as early morning awakenings, with similar prevalences in frequent and infrequent users. None of the controls reported problems with sleep onset, sleep maintenance, or early awakening.

Both insomnia groups scored significantly higher than controls on rating scales of depression (BDI *p* < 0.001; SCL90-R *p* < 0.004), without significant differences between the frequent and infrequent users. Anxiety was also increased in insomniacs as compared to controls, as shown by significantly higher scores on both subscales of the STAI in both groups (STAI state: frequent users *p* = 0.044 and infrequent users *p* < 0.001; STAI trait: frequent users *p* = 0.005 and infrequent users *p* < 0.001). A significant difference on the SCL90-R anxiety scale was found between the infrequent users and controls (*p* = 0.006), but not between the frequent users and controls. There were no significant differences in anxiety ratings between the insomnia groups.

The Multidimensional Fatigue Inventory showed significant differences between groups on all five subscales. Both insomnia groups reported suffering from significantly more general fatigue (both *p* < 0.001), physical fatigue (both *p* < 0.001), and more mental fatigue (frequent users *p* < 0.001 and infrequent users *p* = 0.002) when compared to the control group. In addition, both groups reported significant reductions in motivation (frequent users *p* = 0.015 and infrequent users *p* = 0.027), and frequent users also reported reduced activity (*p* = 0.040). No differences between the insomnia groups were found on any of the scales.

### Sleep diary

The 2-week sleep diary showed that complaints of disturbed sleep as measured by the GSQS-specific were significantly increased in both insomnia groups as compared to controls (Table 2). Compared with the healthy, good sleepers, the infrequent users group reported significantly more sleep complaints (*p* < 0.001), longer sleep onset time (*p* < 0.01), shorter total sleep time (*p* < 0.001), reduced sleep efficiency (*p* < 0.001), earlier morning awakenings (*p* < 0.05), and more awakenings (*p* < 0.001). Sleep quality in the frequent users group was significantly worse as compared to the good sleepers in number of sleep complaints (*p* < 0.001), sleep onset time (*p* < 0.01), and sleep efficiency (*p* < 0.01). Comparisons between the two insomnia groups revealed that the infrequent users reported a shorter total sleep time (*p* < 0.01), the lower sleep efficiency (*p* < 0.01), and more awakenings (*p* < 0.05) than the frequent users showing that sleep was worst in infrequent user group.

**Table 2:** Sleep diary

				Main effect of Group^a^	
Variable	Frequent users	Infrequent users	Controls	*F*	*p*
Groningen Subjective Quality of Sleep scale	6.0 (2.6)^b^	6.8 (2.0)^b^	1.9 (1.1)	56.40	<0.001
Time in bed (min)	521 (61)	499 (53)	495 (37)	1.37	0.267
Total sleep time (min)	411 (73)	349 (71)^b, c^	440 (37)	12.53	<0.001
Sleep efficiency (%)	79 (13)^b^	69 (12)^b, c^	89 (7)	21.87	<0.001
Sleep onset time (min)	43 (37)^b^	44 (30)^b^	17 (18)	7.96	0.001
Number of awakenings	0.8 (0.7)	1.4 (0.9)^b, c^	0.5 (0.4)	7.77	0.002
Early morning awakening (min)	33 (43)	50 (42)^b^	18 (14)	5.85	0.007

^a^Using the Welch test of one-way ANOVA

^b^Significant difference with control group (*p* < 0.05; post hoc analysis with LSD correction)

^c^Significant difference between frequent and in frequent users (*p* < 0.05; post hoc analysis with LSD correction)

### Hypnotic use

The hypnotics used in the frequent users group were zopiclone (*n* = 4), temazepam (*n* = 4), midazolam (*n* = 4), oxazepam (*n* = 3), zolpidem (*n* = 2), lormetazepam (*n* = 2), clonazepam (*n* = 1), flurazepam (*n* = 1), and nitrazepam (*n* = 1). Their average (±SD) duration of hypnotic use was 7.7 (6.8) years. The average (±SD) nightly use of the hypnotics was 6.4 (1.2) nights a week (Table 3).

**Table 3:** Overview of individual hypnotic use and their pharmacokinetic properties. Hypnotics are listed in increasing order of expected residual effects

Hypnotic	Dose (mg)	*t*_1/2_ (hours)^a^	Residual effects 8–12 h post dose^b^	SDLP (in cm)	Duration of use (years)	Nights per week	Sex	Age
Zolpidem	10	1.9 ± 0.2	I (unlikely)	12.5	3	4	Female	58
Zolpidem	10	1.9 ± 0.2	I (unlikely)	15.6	10	7	Male	59
Midazolam	7.5	1.9 ± 0.6	I (unlikely)	13.2	1.5	7	Male	68
Midazolam	7.5	1.9 ± 0.6	I (unlikely)	16.8	5	7	Male	64
Midazolam	7.5	1.9 ± 0.6	I (unlikely)	16.3	4	7	Male	64
Midazolam	7.5	1.9 ± 0.6	I (unlikely)	19.9	3	7	Female	57
Lormetazepam	0.5	10 ± 2.5	I (unlikely)	20.4	7	6	Male	55
Temazepam	10	11	I (unlikely)	14.7	30	4	Female	63
Temazepam	10	11	I (unlikely)	27.6	4	4	Male	65
Temazepam	10	11	I (unlikely)	24.0	15	7	Female	69
Temazepam	20	11	I (unlikely)	13.3	1.5	7	Female	56
Zopiclone	3.75	5	I (unlikely)	12.3	3	7	Female	58
Zopiclone	3.75	5	I (unlikely)	12.8	15	7	Female	63
Zopiclone	3.75	5	I (unlikely)	18.9	2	7	Female	60
Nitrazepam	5	26 ± 3	II (minor)	21.6	12	7	Female	68
Zopiclone	7.5	5	II (moderate)	17.0	13	7	Male	61
Oxazepam	10	8 ± 2.4	NA	16.5	1.5	7	Male	68
Oxazepam	20	8 ± 2.4	NA	15.4	9	7	Male	62
Oxazepam	50	8 ± 2.4	II (moderate)	25.2	1	7	Male	63
Lormetazepam	2	10 ± 2.5	II (moderate)	16.0	10	4	Male	63
Flurazepam	15	1–2 (74 ± 24)^c^	II (moderate)	14.1	11	7	Female	56
Clonazepam	0.5	19–60	NA	18.5	7	7	Female	67

^a^Information derived from Vermeeren (2004), information about clonazepam derived from (Riss et al. 2008)

^b^Hypnotics’ residual effects are categorized according to a calibration scheme in which the impairment of a hypnotic is compared with blood alcohol concentrations (BAC). Category I = unlikely to produce an effect, equivalent to BAC <0.2 g/L; category II = likely to produce minor or moderate effects, equivalent to BAC 0.2–0.5 g/L; category III = likely to produce severe effects, equivalent to BAC >0.5 g/L (Wolschrijn et al. 1991; De Gier et al. 2009); NA = information not available

^c^Half-life of active metabolite between brackets

Within the infrequent users group, seven patients reported no history of hypnotic use. The remaining 13 patients used hypnotics infrequently and irregularly. Their average (±SD) nightly use was 4.1 (2.9) nights a month and with an average (±SD) duration of hypnotic use of 7.8 (7.9) years. The hypnotics used were temazepam (*n* = 6), zopiclone (*n* = 4), lorazepam (*n* = 1), loprazolam (*n* = 1), and nitrazepam (*n* = 1).

### Sleep the night before driving

Table 4 presents the sleep parameters of the objective and subjective sleep evaluation for each group. Polysomnography showed no differences between the three groups on any of the parameters measured. In contrast, significant overall group differences were found for subjective evaluations of total sleep time (*p* = 0.005), sleep onset time, and sleep quality (*p* = 0.001). Both insomnia groups reported significantly more sleep complaints on the GSQS than the control group (*p* values <0.01). In addition, the infrequent users group reported significantly shorter total sleep times (*p* < 0.01) and longer sleep onset times (*p* < 0.05) than the control group. The frequent users group did not differ from the control group.

**Table 4:** Mean (±SD) of objective and subjective sleep parameters

				Main effect of Group^a^	
Variable	Frequent users	Infrequent users	Controls	*F*	*p*
Total sleep time (minutes)					
Polysomnography	383 (35)	389 (46)	408 (40)	2.34	0.107
Subjective evaluation	351 (101)	302 (94)^b^	395 (74)	6.21	0.005
Sleep onset time (minutes)					
Polysomnography	26 (10)	19 (13)	19 (15)	2.83	0.071
Subjective evaluation^c^	27 (16)	68 (73)^b, d^	35 (37)	4.18	0.023
Number of awakenings					
Polysomnography	7.9 (4.1)	10.2 (4.3)	7.5 (4.5)	2.33	0.111
Subjective evaluation^c^	2.1 (1.6)	3.0 (2.5)	1.9 (1.5)	0.99	0.382
Polysomnographic parameters					
Sleep efficiency (%)	80 (9)	81 (9)	85 (8)	1.89	0.165
Wake after sleep onset (minutes)	77 (65)	73 (39)	55 (36)	1.53	0.229
Stage 1 sleep (% of total sleep time)	7 (3)	7 (2)	6 (3)	0.72	0.492
Stage 2 sleep (% of total sleep time)	57 (6)	55 (7)	56 (9)	0.66	0.522
Stage SWS sleep (% of total sleep time)	18 (9)	19 (6)	17 (6)	0.67	0.517
Stage REM sleep (% of total sleep time)	19 (4)	20 (6)	22 (6)	1.84	0.173
Subjective evaluation					
Early morning awakening (minutes)^c^	51 (58)	67 (62)	28 (35)	2.52	0.093
Groningen Subjective Quality of Sleep scale	6.7 (3.9)^b^	7.8 (3.8)^b^	3.6 (3.2)	8.48	0.001

^a^Using the Welch test of one-way ANOVA

^b^Significant difference with control group (*p* < 0.05; post hoc analysis with LSD correction)

^c^Scores were transformed (ln) for statistical analysis to correct for skewness

^d^Significant difference between frequent and in frequent users (*p* < 0.05; post hoc analysis with LSD correction)

### Driving, cognitive, and psychomotor performance

**Fig. 1:**
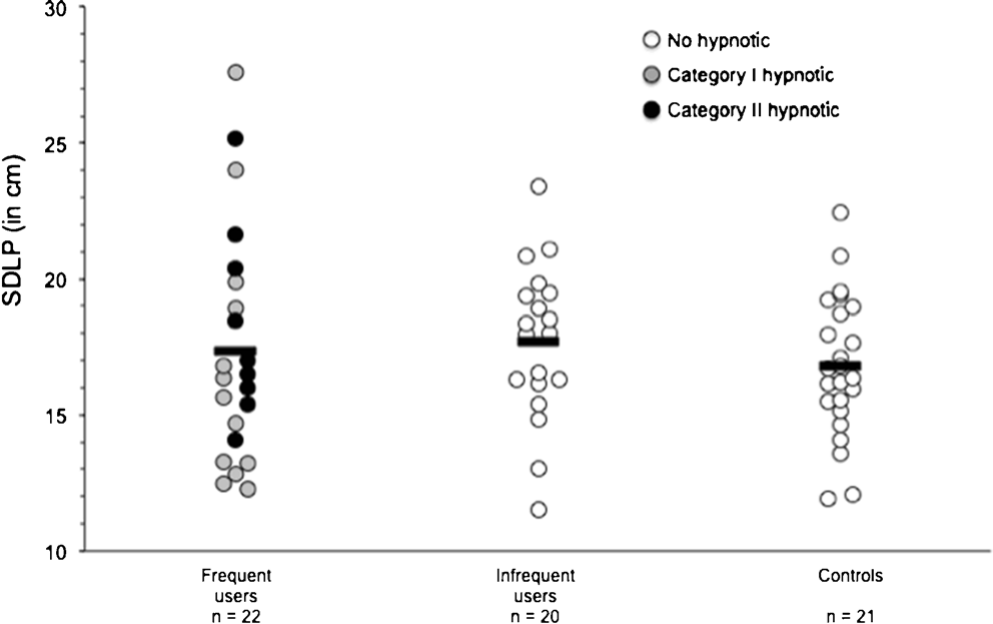
Individual SDLP data for each group separately. *Horizontal bars* indicate average SDLP

Figure 1 and Table 5 show the driving performance parameters. The primary performance parameter, SDLP in the highway driving test, was on average normal and similar for infrequent users (17.7 ± 2.9 cm), frequent users (17.4 ± 4.3 cm), and controls (16.8 ± 2.7 cm) (Fig. 1). Table 3 illustrates the hypnotics and doses used by the frequent users and the corresponding SDLP scores. Inspection of the average SDLP scores from users of category I drugs and category II drugs shows that performance in the category I group appears to be better than the category II group. Mean (±SD) SDLP scores are 16.8 (4.7) and 18.3 (3.5) cm, respectively.

**Table 5:** Mean (±SD) of driving performance parameters

				Main effect of Group^a^	
Variable	Frequent users	Infrequent users	Controls	*F*	*p*
Highway Driving Test					
Standard deviation of lateral position (in cm)	17.4 (4.3)	17.7 (2.9)	16.8 (2.7)	0.74	0.484
Mean speed (in km/h)	94.5 (1.5)	93.9 (1.9)	94.3 (1.4)	0.60	0.555
Standard deviation of speed (in km/h)	2.11 (0.51)	2.34 (0.68)	2.18 (0.66)	1.01	0.371
Car-Following Test					
Reaction time (in s)	3.55 (1.57)	3.32 (1.40)	3.06 (1.01)	0.10	0.906
Gain	1.14 (0.13)	1.21 (0.25)	1.15 (0.12)	0.46	0.637
Subjective Evaluation of Driving Test					
Subjective driving quality	67 (11)	68 (10)	65 (14)	0.73	0.488
Apparent sedation	12 (11)	12 (15)	9 (10)	1.01	0.349
Mental effort^b^	30 (23)	36 (25)	20 (14)	2.52	0.090

^a^ANCOVA with sex, age, and education as covariates

^b^Scores were transformed (ln) for statistical analysis to correct for skewness

The secondary driving task, the Car-Following Test, was not different between the groups as well. All participants were able to respond to the changes of the preceding car in a similar fashion.

Finally, there were no significant differences in performance on any of the psychomotor or cognitive tests. There were tendencies (*p* < 0.10) towards group differences in the divided attention test and the digit span test. For the digit span test, this was mainly due to worse performance by frequent users as compared to controls in the morning. In the divided attention test, there was a tendency for group differences in the tracking subtask, due to worse tracking performance by the controls as compared to the both patient groups.

Comparisons between performance in the evening and morning sessions showed significant differences in the reaction times in the PVT and delayed recall in the word learning test. The latter was as expected because the interval between learning and delayed recall increased from evening to morning sessions. Average reaction time in the PVT was significantly faster in the morning as compared to the evening in controls (*p* < 0.005), but not in the insomnia groups.

Performance in the critical tracking test, divided attention task, and the stop signal task showed no significant differences between times of day (evening vs. morning).

### Subjective rating scales

As shown in Table 6, patients felt on average less alert and more sleepy than controls as measured by the mood rating scale and the KSS, but this was only significant for alertness in the evening (*p* = 0.002). In the evening, frequent users had significantly lower alertness scores than controls. Furthermore, all groups felt on average less alert and more sleepy in the morning as compared to the evening. Pairwise comparisons showed that these differences were only significant in the controls (*p* < 0.01), but not in the patients.

**Table 6:** Mean (±SD) of psychomotor and cognitive performance parameters

				Main effect of Group^a^	
Variable	Frequent users (*n* = 22)	Infrequent users (*n* = 20)	Controls (*n* = 21)	*F*	*p*
Critical Tracking Task					
Average lambda (in rad/s), evening	3.34 (0.72)	3.23 (0.60)	3.03 (0.49)	2.39	0.101
Average lambda (in rad/s), morning	3.29 (0.70)	3.21 (0.72)	3.02 (0.49)	1.26	0.293
Divided Attention Task					
Tracking subtask: average error (in mm), evening	14.1 (5.2)	14.4 (4.4)	17.4 (5.2)	2.45	0.095
Tracking subtask: average error (in mm), morning	15.5 (5.9)^b^	14.9 (4.2)^b^	18.7 (5.2)	2.87	0.065^c^
Detection subtask: reaction time (in ms), evening	1,924 (328)	1,974 (299)	1,973 (335)	0.70	0.501
Detection subtask: reaction time (in ms), morning	2,030 (344)	1,898 (288)	1,920 (338)	0.33	0.720
Stop Signal Task					
Go reaction time (in ms)	423 (63)	427 (77)	422 (50)	0.98	0.907
Stop signal reaction time (in ms)	179 (35)	178 (31)	181 (35)	0.02	0.980
Psychomotor Vigilance Task					
Median reaction time (in ms), evening	254 (34)	244 (31)	247 (23)	0.47	0.631
Median reaction time (in ms), morning	252 (35)	242 (30)	241 (21)	1.52	0.228
Lapses (>500 ms), evening	1.2 (2.0)	1.3 (1.1)	1.0 (1.3)	0.12	0.885
Lapses (>500 ms), morning	1.3 (2.1)	0.7 (1.0)	0.7 (1.2)	0.71	0.495
Word Learning Task					
Immediate total recall score	46.3 (12.3)	46.8 (9.4)	49.4 (9.0)	0.35	0.707
Delayed recall score, evening	7.8 (3.5)	8.4 (2.9)	8.9 (3.4)	1.36	0.266
Delayed recall score, morning	6.5 (2.5)	7.0 (3.4)	7.3 (3.2)	0.72	0.491
Recognition score	24.8 (4.4)	24.6 (3.0)	25.7 (3.4)	0.30	0.746
Recognition reaction time (in ms)	924 (189)	892 (192)	883 (141)	0.14	0.868
Digit Span					
Forward score, evening	3.6 (1.3)	3.8 (1.1)	3.9 (1.2)	0.37	0.693
Forward score, morning	3.5 (0.9)^b^	4.0 (1.1)	4.4 (1.1)	2.88	0.064^c^
Backward score, evening	3.5 (1.3)	3.9 (1.4)	4.3 (1.1)	1.61	0.209
Backward score, morning	3.3 (1.0)^b^	3.8 (1.4)	4.2 (1.1)	2.66	0.079^c^
Subjective Evaluations of Feelings					
Alertness, evening	66 (17)^*, b^	74 (13)	81 (13)	6.80	0.002^c^
Alertness, morning	64 (19)	69 (14)	74 (15)	2.86	0.066
Contentedness, evening	73 (21)	78 (13)	83 (13)	2.19	0.122
Contentedness, morning	75 (19)	76 (12)	81 (14)	1.02	0.368
Calmness, evening	70 (23)	75 (14)	82 (14)	2.64	0.081
Calmness, morning	73 (17)	73 (14)	80 (14)	0.93	0.401
Karolinska Sleepiness Scale, evening	3.91 (1.66)^b^	3.90 (1.65)^b^	3.00 (0.84)	2.90	0.063^c^
Karolinska Sleepiness Scale, morning	4.55 (1.50)	4.30 (1.72)	3.81 (1.25)	1.86	0.165

^*^ *p* < 0.05, significant difference from control group using pairwise comparisons of ANCOVA

^a^ANCOVA with sex, age, and education as covariates; ;

^b^Significant difference with control group (*p* < 0.05; post hoc analysis with LSD correction)

^c^Significant (*p* < 0.05) main effect of group using the Welch test of one-way ANOVA

No group differences were found in the evaluation by the instructors of the participants’ driving quality and apparent sedation. In addition, although showing a marginal trend to the detriment of the infrequent users, no differences between the groups were found in subjectively rated mental effort the participants needed to put in the driving test.

## Discussion

The present study is the first directly comparing driving performance between insomnia patients who chronically use hypnotics, insomnia patients who do not or infrequently use hypnotics, and healthy, good sleepers. Results show that driving, as measured by a standardized highway driving test and a car-following test, is not impaired in insomniacs, irrespective of the use of hypnotics. In addition, the present study shows that driving-related psychomotor and cognitive performance is not different between pharmacologically treated insomnia patients, untreated insomnia patients, and healthy, good sleepers.

Results of the study corroborate previous findings showing an absence of neuropsychological deficits in both pharmacologically treated and untreated insomniacs (Vignola et al. 2000). Aside from minor attentional problems in insomniacs, that study reported no significant differences in performance between treated insomniacs, untreated insomniacs, and healthy, good sleepers. The authors suggested, however, that the absence of cognitive impairment may have been due to methodological limitations. The tests used in their study were of short duration and demanded low effort (e.g., digit symbol substitution test and purdue pegboard test). Consequently, insomnia patients may have been able to exert enough effort for a short period to complete the tests successfully.

In the present study, subjects performed a standardized highway driving test for approximately 1 h. The prolonged attentional demands of the task were expected to reveal possible performance deficits in insomnia patients, which were not found with tasks of short duration. Yet, there were no indications of deterioration in driving performance in insomnia patients, irrespective of use of hypnotics.

These results can be interpreted in two ways. First, sleep in the insomnia groups was undisturbed according to polysomnographic criteria leaving daytime performance unaffected. Despite significantly more subjective sleep complaints reported by the insomniacs when compared with the healthy, good sleepers, there was no objective evidence for any sleeping problems. Polysomnographic data showed that there were no differences between the groups on any of the sleep parameters obtained from the laboratory recordings. Discrepancies between subjective and objective sleep parameters have been established in previous studies (e.g., Orff et al. 2007; Vignola et al. 2000). A limitation in those studies was that participants’ sleep was only assessed during one night, possibly causing “first night effects” in healthy, good sleepers and “reversed first night effects” in insomnia patients. The present study, therefore, used the recommended second night recordings for determination of objective sleep complaints. Yet, there were no indications of any objective evidence for sleeping difficulties. It is suggested, however, that the current standards of sleep analysis may not have sufficient sensitivity for distinguishing insomnia from healthy, undisturbed sleep (Bastien et al. 2003). A possible solution can be found in spectral analysis of the sleep microstructure, dissociating characteristic electroencephalographic activities. More research is needed to confirm this presumption.

The lack of objectively measured sleep complaints may be also due to the sleeping environment. It has been shown that home-based polysomnographic recordings yield different results than recordings at the laboratory (Edinger et al. 1997). Insomnia patients’ sleep appears more disturbed when they sleep at home than when they sleep at the laboratory. Indeed, subjective sleep evaluations in the present study show that sleep quality at the laboratory improved in both insomnia groups. In addition, subjective sleep quality for the healthy controls was worse after laboratory sleep than after sleep at home. Sleep quality changed even close to insomnia levels on some of the parameters. These changes in sleep quality may have resulted in a less pronounced difference between the groups and may have minimized existing differences in objective sleep recordings. Additionally, the changed sleep quality may have had an influence on daytime performance, i.e., improved in patients due to improved sleep and impaired in healthy controls due to impaired sleep. Improved daytime functioning as a result of improved sleep in insomnia patients has been shown previously (Edinger et al. 2003). In contrast, healthy good sleepers, in particular elderly, do not seem to suffer greatly from a single night of disturbed sleep (Philip et al. 2004). Still, it remains to be investigated whether sleep at home would influence driving performance differently than sleep at the laboratory in both insomnia patients and good sleepers.

A second explanation for the absence of impairment in driving performance may be that the insomnia patients, who had ample driving experience, may have been easily able to complete the driving tests successfully. In a recent study, it has been shown that establishing performance impairment in insomnia may be dependent on task complexity (Altena et al. 2008). Insomnia patients performed worse than healthy controls only in a complex vigilance task, whereas the patients’ performance in a simple reaction time task appeared to be even better than healthy controls. The authors concluded that chronic insomnia is associated with cognitive dysregulation, but that this may only be revealed in tasks measuring higher level functioning. Driving is a well-practiced and highly automated skill (Brouwer 2002) and may not require such high demands on cognitive functioning. Judging from the low values scored on the mental effort scale, this assumption seems to be confirmed. Scores on the scale can range from 0 to 150. The average scores in the present study were 30.3 for the medicated insomnia patients, 35.7 for the unmedicated insomnia patients, and 20.3 for the healthy controls. In a study comparing cognitive performance between patients with seasonal allergic rhinitis and healthy controls, subjects evaluated the mental effort they had to put in a 45-min sustained attention test considerably higher (Hartgerink-Lutgens et al. 2009). Mental effort scores were around 90 for both groups, indicating substantial higher demands of that test as compared with the highway driving test.

Yet, the insomniacs, in particular the infrequent users group, evaluated the degree of mental effort they had to put in the driving test tentatively higher than the healthy controls. Although not reaching statistical significance, this may suggests that the insomnia patients masked possible performance difficulties by increasing their effort. As mentioned earlier, the short duration of cognitive tasks was considered to be a limitation for revealing any performance deficits in insomnia patients. It seems that even tasks with duration of at least 1 h can be completed successfully by individuals reporting sleeping difficulties.

In addition to the findings that driving is not affected in insomnia patients, results of the present study show that driving is not impaired in patients chronically using hypnotics as well. The absence of impairment, combined with the still present subjective sleep complaints, suggests the development of tolerance to both therapeutic and residual effects of hypnotics. With respect to the residual effects, the results are partly supported by epidemiological data (Neutel 1998). These showed that prolonged use of hypnotics is associated with a lowered risk of becoming involved in a car accident when compared with initial use of hypnotics. Nevertheless, the risk of injurious traffic accidents after chronic use of hypnotics remained twice as high in long-term hypnotic users in comparison to healthy, unmedicated drivers.

The absence of residual effects in the present study may be explained by the wide variety of hypnotic drugs and doses used in the frequent users group, however. In addition, the majority of hypnotics were unlikely to produce residual effects between 10 to 11 h post dose. Consequently, the large differences in degree of residual effects may have contributed to the variability of performance in this group and masked any detectable impairment. This is supported by the inspection of the average SDLP scores from the category I and the category II users, showing that the former group had a slightly lower average SDLP score (16.8 cm) than the latter (18.3 cm). Comparing these values with two studies investigating the residual effects of zopiclone 7.5 mg in younger (Leufkens et al. 2009) and older (Leufkens and Vermeeren 2009) healthy, medication naïve, participants reveals that performance was in the range of driving after administration of placebo. Both age groups had placebo SDLP scores of 17.8 cm (95 % confidence interval 16.8–18.2 cm). Future research in patients chronically using the same hypnotic is needed to shed more light on this issue. The present study aimed, however, to evaluate driving performance in a representative, non-selective study sample of insomnia patients chronically using hypnotics.

The results may differ when driving is investigated in a younger sample of insomniacs. A recent review showed that the residual effect of zopiclone on driving in older subjects (age ranging from 56 to 73) was generally less than that found in younger subjects (age ranging from 21 to 45 years) (Leufkens and Vermeeren 2013). An explanation for this effect may be found in age-related increases in driving experience. The driving-related skills of less experienced drivers may be more sensitive to drug-induced sedation. Consequently, driving performance could be impaired to a larger extent by hypnotic use in younger insomnia patients than in older patients.

It may be argued that the driving tests are not sensitive enough to detect performance deficits as a consequence of a disorder. Studies assessing driving performance in other patient groups using the same standardized driving test have been conducted previously. Direct comparisons with healthy controls have been reported for patients with chronic nonmalignant pain (Veldhuijzen et al. 2006) and depressed patients receiving long-term antidepressant treatment (Wingen et al. 2006). In contrast to the present study, these studies showed that driving was significantly impaired in both patient groups as compared with healthy controls. These results suggest that the driving test has sufficient sensitivity for detecting driving impairment in patient groups. In addition, sample sizes of the previous studies were similar to the present study, suggesting that the absence of effects of insomnia could not be explained in terms of statistical power.

Finally, it may be questioned whether the study had sufficient power to detect differences in driving performance between insomnia patients and controls. We do not believe this is the case. First of all, the mean difference in SDLP between infrequent users and controls was small (i.e., less than 1 cm), and not considered clinically relevant. The minimum clinically relevant difference in SDLP is 2.4 cm which corresponds to the effect of alcohol when blood alcohol concentrations are at the legal limit for driving in most countries. Secondly, previous studies assessing driving performance in other patients groups, using the same standardized driving test, have shown that the method is sufficiently sensitive to detect significant impairment in small samples of patients with chronic nonmalignant pain (Veldhuijzen et al. 2006) and depressed patients receiving long-term antidepressant treatment (Wingen et al. 2006). It seems therefore that the effects of insomnia and chronic use of hypnotics are relatively small and less debilitating than the effects of pain and depression.

To conclude, results of the present study indicate that driving performance is not substantially impaired in older chronic users of hypnotics and older insomnia patients who do not use hypnotics. This supports conclusions from previous studies that effects of insomnia and chronic use of hypnotics on cognitive performance are subtle.
